# (*E*)-3-(2-Chloro­phen­yl)-1-(2,4-dichloro­phen­yl)prop-2-en-1-one

**DOI:** 10.1107/S160053680801413X

**Published:** 2008-05-17

**Authors:** Hoong-Kun Fun, Suchada Chantrapromma, P. S. Patil, S. M. Dharmaprakash

**Affiliations:** a X-ray Crystallography Unit, School of Physics, Universiti Sains Malaysia, 11800 USM, Penang, Malaysia; b Department of Chemistry, Faculty of Science, Prince of Songkla University, Hat-Yai, Songkhla 90112, Thailand; c Department of Studies in Physics, Mangalore University, Mangalagangotri, Mangalore 574 199, India

## Abstract

In the title chalcone derivative, C_15_H_9_Cl_3_O, the dihedral angle between the 2-chloro­phenyl and 2,4-dichloro­phenyl rings is 41.79 (14)°. Weak C—H⋯O and C—H⋯Cl intra­molecular inter­actions involving the enone unit generate *S*(5) ring motifs. In the crystal structure, the mol­ecules are arranged in a head-to-tail manner along the *a* axis. These chains are stacked along the *b* axis.

## Related literature

For related literature on hydrogen-bond motifs, see: Bernstein *et al.* (1995 ▶). For bond-length data, see: Allen *et al.* (1987 ▶). For related structures, see, for example: Fun, Chantrapromma *et al.* (2007 ▶); Fun, Patil *et al.* (2007 ▶); Patil, Chantrapromma *et al.* (2007 ▶; Patil, Fun *et al.* (2007 ▶). For background to the applications of substituted chalcones, see, for example: Agrinskaya *et al.* (1999 ▶); Gu *et al.* (2008 ▶); Patil, Dharmaprakash *et al.* (2007 ▶).
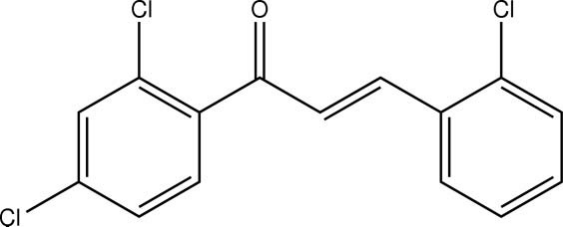


## Experimental

### 

#### Crystal data

C_15_H_9_Cl_3_O
*M* *_r_* = 311.57
Monoclinic, 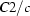

*a* = 50.177 (2) Å
*b* = 3.8082 (2) Å
*c* = 13.7297 (7) Å
β = 95.307 (3)°
*V* = 2612.3 (2) Å^3^
*Z* = 8
Mo *K*α radiation
μ = 0.69 mm^−1^
*T* = 100.0 (1) K
0.39 × 0.20 × 0.14 mm

#### Data collection

Bruker SMART APEX2 CCD area-detector diffractometer
Absorption correction: multi-scan (**SADABS**; Bruker, 2005 ▶) *T*_min_ = 0.775, *T*_max_ = 0.910
13605 measured reflections
2976 independent reflections
2374 reflections with *I* > 2σ(*I*)
*R*_int_ = 0.054

#### Refinement

*R*[*F* ^2^ > 2σ(*F* ^2^)] = 0.066
*wR*(*F* ^2^) = 0.189
*S* = 1.13
2976 reflections
172 parameters
H-atom parameters constrained
Δρ_max_ = 0.52 e Å^−3^
Δρ_min_ = −0.58 e Å^−3^

### 

Data collection: *APEX2* (Bruker, 2005 ▶); cell refinement: *APEX2*; data reduction: *SAINT* (Bruker, 2005 ▶); program(s) used to solve structure: *SHELXTL* (Sheldrick, 2008 ▶); program(s) used to refine structure: *SHELXTL*; molecular graphics: *SHELXTL*; software used to prepare material for publication: *SHELXTL* and *PLATON* (Spek, 2003 ▶).

## Supplementary Material

Crystal structure: contains datablocks global, I. DOI: 10.1107/S160053680801413X/is2291sup1.cif

Structure factors: contains datablocks I. DOI: 10.1107/S160053680801413X/is2291Isup2.hkl

Additional supplementary materials:  crystallographic information; 3D view; checkCIF report

## Figures and Tables

**Table 1:** Hydrogen-bond geometry (Å, °)

*D*—H⋯*A*	*D*—H	H⋯*A*	*D*⋯*A*	*D*—H⋯*A*
C9—H9*A*⋯Cl3	0.93	2.66	3.042 (5)	106
C9—H9*A*⋯O1	0.93	2.53	2.841 (6)	100

**Fig. 1.**
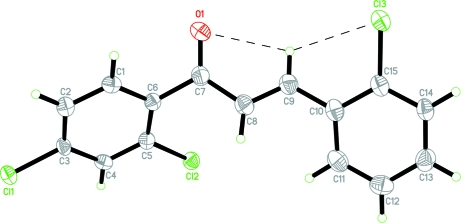
The asymmetric unit of (I), showing 50% probability displacement ellipsoids and the atomic numbering. Weak intramolecular C—H···O and C—H···Cl interactions are drawn as dashed lines.

**Fig. 2.**
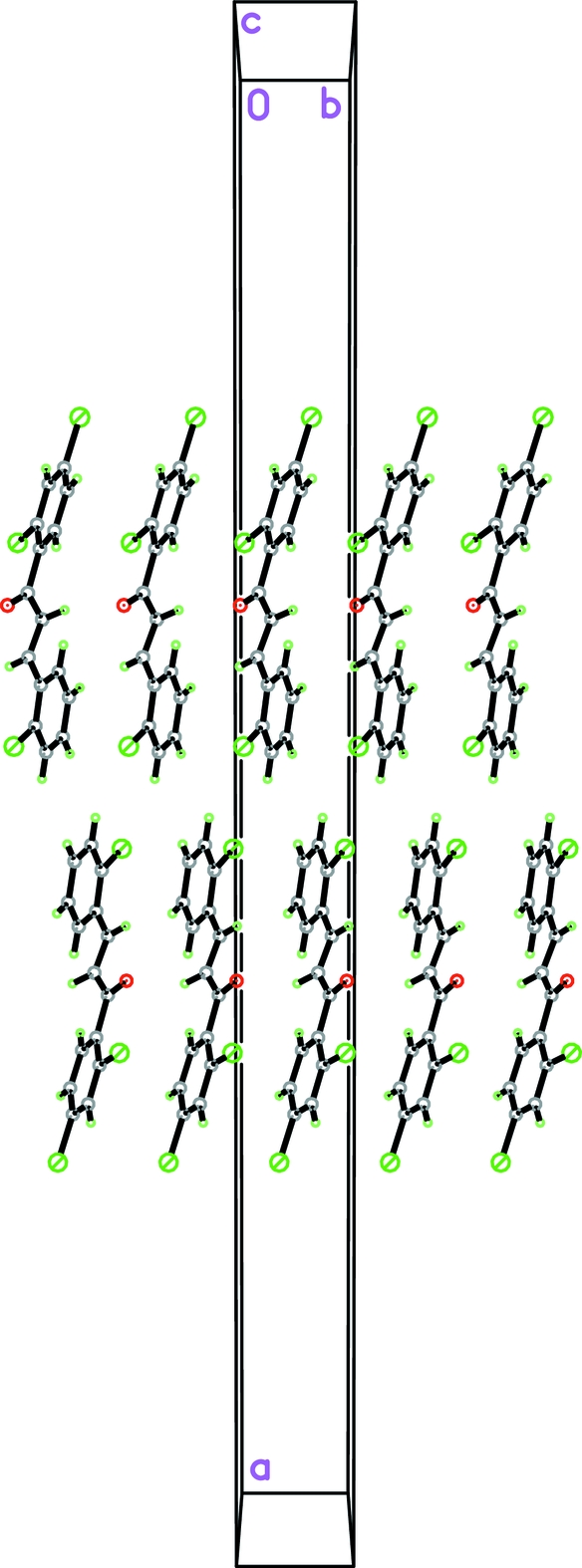
The crystal packing of (I), viewed along the c axis showing head-to-tail arrangement along the a axis and stacking of the molecules along the b axis.

## supplementary crystallographic information

### Comment

Nonlinear optical properties of chalcone derivatives have been widely investigated recently (Agrinskaya *et al.*, 1999; Fun, Chantrapromma *et al.*, 2007; Fun, Patil *et al.*, 2007; Patil, Dharmaprakash *et al.*, 2007; Patil, Chantrapromma *et al.*, 2007; Patil, Fun *et al.*, 2007). These molecules show potential in optical-limiting applications due to their large excited-state absorption cross sections (Gu *et al.*, 2008). In view of the importance of chalcones and the continuation of our non-linear optic materials research the title chalcone derivative, (I), was synthesized and its crystal structure is reported here.

In the structure of the title chalcone derivative (Fig. 1), the enone unit O1/C6–C8, the 2-chlorophenyl and 2,4-dichlorophenyl rings are individually planar, with the maximum deviations of 0.016 (6), -0.017 (6) and 0.022 (5) Å for atom C7, C11 and C2, respectively. The molecule is slightly twisted about the C6–C7 bond as indicated by the torsion angles C1–C6–C7–C8 = 132.8 (5)°, C6–C7–C8–C9 = 171.6 (5)°, C7–C8–C9–C10 = -179.7 (5)° and C8–C9–C10–C15 = -160.7 (5)°. The dihedral angles between the 2-chlorophenyl and 2,4-dichlorophenyl rings is 41.79 (14)°. The least-squares plane through the enone unit makes dihedral angles of 10.3 (3)° and 46.9 (2)° with the 2-chlorophenyl and 2,4-dichlorophenyl rings, respectively. The orientation of the prop-2-en-1-one unit can be indicated by the torsion angle O1–C7–C8–C9 = -11.5 (8)°. Bond lengths and angles in (I) are in normal ranges (Allen *et al.*, 1987) and comparable to those in related structures (Fun, Chantrapromma *et al.*, 2007; Fun, Patil *et al.*, 2007; Patil, Dharmaprakash *et al.*, 2007; Patil, Chantrapromma *et al.*, 2007; Patil, Fun *et al.*, 2007).

In the molecular structure, both weak C9—H9A···O1 and C9—H9A···Cl1 intramolecular interactions (Table 1) generate S(5) ring motifs (Bernstein *et al.*, 1995). In the crystal structure (Fig. 2), the molecules are arranged in a head-to-tail manner along the *a*-axis. These chains are stacked along the *b* axis.

### Experimental

The title compound was synthesized by the condensation of 2-chlorobenzaldehyde (0.01 mol) with 2,4-dichloroacetophenones (0.01 mol) in methanol (60 ml) in the presence of a catalytic amount of sodium hydroxide solution (5 ml, 30%). After stirring (4 h), the contents of the flask were poured into ice-cold water (500 ml) and left to stand for 6 h. The resulting crude solid was filtered and dried. Colorless block-shaped single crystals of the title compound suitable for X-ray structure determination were recrystallized from acetone by slow evaporation of the solvent at room temperature.

### Refinement

All H atoms were placed in calculated positions (C—H = 0.93 Å) and treated as riding, with *U*_iso_(H) = 1.2*U*_eq_(C). The highest residual electron density peak is located at 1.90 Å from C13 and the deepest hole is located at 0.93 Å from Cl2.

### Crystal data

**Table:**

C_15_H_9_Cl_3_O	*F*_000_ = 1264
*M**_r_* = 311.57	*D*_x_ = 1.584 Mg m^−^^3^
Monoclinic, *C*2/*c*	Mo *K*α radiation λ = 0.71073 Å
Hall symbol: -C 2yc	Cell parameters from 2976 reflections
*a* = 50.177 (2) Å	θ = 0.8–27.5º
*b* = 3.8082 (2) Å	µ = 0.69 mm^−^^1^
*c* = 13.7297 (7) Å	*T* = 100.0 (1) K
β = 95.307 (3)º	Block, colorless
*V* = 2612.3 (2) Å^3^	0.39 × 0.20 × 0.14 mm
*Z* = 8	

### Data collection

**Table:**

Bruker SMART APEX2 CCD area-detector diffractometer	2976 independent reflections
Radiation source: fine-focus sealed tube	2374 reflections with *I* > 2σ(*I*)
Monochromator: graphite	*R*_int_ = 0.054
Detector resolution: 8.33 pixels mm^-1^	θ_max_ = 27.5º
*T* = 100.0(1) K	θ_min_ = 0.8º
ω scans	*h* = −64→64
Absorption correction: multi-scan(*SADABS*; Bruker, 2005)	*k* = −4→4
*T*_min_ = 0.775, *T*_max_ = 0.911	*l* = −17→17
13605 measured reflections	

### Refinement

**Table:**

Refinement on *F*^2^	Secondary atom site location: difference Fourier map
Least-squares matrix: full	Hydrogen site location: inferred from neighbouring sites
*R*[*F*^2^ > 2σ(*F*^2^)] = 0.066	H-atom parameters constrained
*wR*(*F*^2^) = 0.189	*w* = 1/[σ^2^(*F*_o_^2^) + (0.0626*P*)^2^ + 28.0963*P*] where *P* = (*F*_o_^2^ + 2*F*_c_^2^)/3
*S* = 1.13	(Δ/σ)_max_ = 0.001
2976 reflections	Δρ_max_ = 0.52 e Å^−^^3^
172 parameters	Δρ_min_ = −0.58 e Å^−^^3^
Primary atom site location: structure-invariant direct methods	Extinction correction: none

### Special details

**Table:**

Experimental. The low-temperature data was collected with the Oxford Cyrosystem Cobra low-temperature attachment.
Geometry. All e.s.d.'s (except the e.s.d. in the dihedral angle between two l.s. planes) are estimated using the full covariance matrix. The cell e.s.d.'s are taken into account individually in the estimation of e.s.d.'s in distances, angles and torsion angles; correlations between e.s.d.'s in cell parameters are only used when they are defined by crystal symmetry. An approximate (isotropic) treatment of cell e.s.d.'s is used for estimating e.s.d.'s involving l.s. planes.
Refinement. Refinement of *F*^2^ against ALL reflections. The weighted *R*-factor *wR* and goodness of fit *S* are based on *F*^2^, conventional *R*-factors *R* are based on *F*, with *F* set to zero for negative *F*^2^. The threshold expression of *F*^2^ > σ(*F*^2^) is used only for calculating *R*-factors(gt) *etc*. and is not relevant to the choice of reflections for refinement. *R*-factors based on *F*^2^ are statistically about twice as large as those based on *F*, and *R*- factors based on ALL data will be even larger.

### Fractional atomic coordinates and isotropic or equivalent isotropic displacement parameters (Å^2^)

**Table:**

	*x*	*y*	*z*	*U*_iso_*/*U*_eq_	
Cl1	0.24912 (2)	0.3582 (3)	0.13634 (9)	0.0253 (3)	
Cl2	0.32818 (2)	0.9466 (3)	−0.04254 (9)	0.0272 (3)	
Cl3	0.46557 (2)	0.9477 (4)	0.12885 (9)	0.0320 (3)	
O1	0.37362 (7)	0.9696 (11)	0.2294 (3)	0.0353 (9)	
C1	0.32520 (9)	0.5622 (13)	0.2282 (4)	0.0240 (10)	
H1A	0.3364	0.5366	0.2855	0.029*	
C2	0.29887 (9)	0.4535 (13)	0.2272 (4)	0.0247 (10)	
H2A	0.2924	0.3562	0.2825	0.030*	
C3	0.28239 (8)	0.4941 (13)	0.1413 (4)	0.0232 (10)	
C4	0.29124 (9)	0.6481 (12)	0.0592 (3)	0.0225 (10)	
H4A	0.2797	0.6825	0.0030	0.027*	
C5	0.31790 (9)	0.7503 (13)	0.0626 (4)	0.0237 (10)	
C6	0.33539 (9)	0.7072 (13)	0.1473 (3)	0.0226 (10)	
C7	0.36458 (9)	0.8125 (15)	0.1559 (4)	0.0292 (11)	
C8	0.38100 (10)	0.7060 (15)	0.0778 (4)	0.0312 (11)	
H8A	0.3740	0.5518	0.0295	0.037*	
C9	0.40609 (9)	0.8285 (15)	0.0751 (4)	0.0310 (11)	
H9A	0.4126	0.9814	0.1245	0.037*	
C10	0.42374 (10)	0.7390 (14)	0.0006 (4)	0.0298 (11)	
C11	0.41423 (10)	0.6012 (15)	−0.0914 (4)	0.0346 (12)	
H11A	0.3960	0.5603	−0.1046	0.042*	
C12	0.43090 (11)	0.5256 (15)	−0.1619 (4)	0.0357 (12)	
H12A	0.4239	0.4393	−0.2224	0.043*	
C13	0.45816 (11)	0.5780 (15)	−0.1430 (4)	0.0357 (12)	
H13A	0.4695	0.5252	−0.1907	0.043*	
C14	0.46862 (9)	0.7084 (14)	−0.0535 (4)	0.0298 (11)	
H14A	0.4870	0.7433	−0.0406	0.036*	
C15	0.45143 (9)	0.7860 (13)	0.0163 (4)	0.0263 (10)	

### Atomic displacement parameters (Å^2^)

**Table:**

	*U*^11^	*U*^22^	*U*^33^	*U*^12^	*U*^13^	*U*^23^
Cl1	0.0192 (5)	0.0235 (6)	0.0333 (6)	−0.0020 (4)	0.0024 (4)	0.0004 (5)
Cl2	0.0291 (6)	0.0242 (6)	0.0288 (6)	−0.0030 (5)	0.0058 (4)	0.0011 (5)
Cl3	0.0250 (6)	0.0343 (7)	0.0363 (7)	−0.0035 (5)	0.0016 (5)	0.0009 (6)
O1	0.0274 (17)	0.042 (2)	0.036 (2)	−0.0053 (16)	−0.0011 (14)	−0.0058 (18)
C1	0.024 (2)	0.021 (2)	0.027 (2)	0.0057 (18)	−0.0006 (17)	−0.004 (2)
C2	0.024 (2)	0.019 (2)	0.032 (3)	0.0036 (18)	0.0049 (18)	0.000 (2)
C3	0.0156 (19)	0.024 (2)	0.031 (2)	−0.0004 (17)	0.0051 (17)	−0.004 (2)
C4	0.024 (2)	0.017 (2)	0.025 (2)	0.0020 (17)	−0.0019 (17)	−0.0009 (19)
C5	0.025 (2)	0.018 (2)	0.028 (2)	0.0008 (18)	0.0052 (18)	0.000 (2)
C6	0.021 (2)	0.021 (2)	0.026 (2)	0.0021 (18)	0.0032 (17)	0.000 (2)
C7	0.026 (2)	0.032 (3)	0.029 (3)	−0.001 (2)	0.0028 (19)	0.002 (2)
C8	0.026 (2)	0.032 (3)	0.035 (3)	0.001 (2)	0.001 (2)	0.000 (2)
C9	0.027 (2)	0.033 (3)	0.033 (3)	0.001 (2)	0.002 (2)	0.002 (2)
C10	0.027 (2)	0.028 (3)	0.035 (3)	−0.001 (2)	0.004 (2)	0.008 (2)
C11	0.032 (3)	0.031 (3)	0.041 (3)	−0.005 (2)	0.000 (2)	0.006 (2)
C12	0.042 (3)	0.026 (3)	0.038 (3)	0.000 (2)	−0.003 (2)	−0.001 (2)
C13	0.034 (3)	0.031 (3)	0.043 (3)	0.006 (2)	0.007 (2)	−0.002 (3)
C14	0.022 (2)	0.028 (3)	0.040 (3)	0.002 (2)	0.0045 (19)	0.002 (2)
C15	0.025 (2)	0.019 (2)	0.035 (3)	−0.0010 (19)	0.0021 (19)	0.006 (2)

### Geometric parameters (Å, °)

**Table:**

Cl1—C3	1.743 (4)	C8—C9	1.347 (7)
Cl2—C5	1.746 (5)	C8—H8A	0.9300
Cl3—C15	1.751 (5)	C9—C10	1.454 (7)
O1—C7	1.224 (6)	C9—H9A	0.9300
C1—C6	1.380 (7)	C10—C15	1.398 (6)
C1—C2	1.383 (6)	C10—C11	1.410 (8)
C1—H1A	0.9300	C11—C12	1.367 (8)
C2—C3	1.385 (7)	C11—H11A	0.9300
C2—H2A	0.9300	C12—C13	1.383 (7)
C3—C4	1.380 (7)	C12—H12A	0.9300
C4—C5	1.390 (6)	C13—C14	1.382 (8)
C4—H4A	0.9300	C13—H13A	0.9300
C5—C6	1.400 (6)	C14—C15	1.380 (7)
C6—C7	1.512 (6)	C14—H14A	0.9300
C7—C8	1.468 (7)		
			
C6—C1—C2	122.4 (4)	C7—C8—H8A	119.6
C6—C1—H1A	118.8	C8—C9—C10	124.7 (5)
C2—C1—H1A	118.8	C8—C9—H9A	117.6
C1—C2—C3	118.0 (4)	C10—C9—H9A	117.6
C1—C2—H2A	121.0	C15—C10—C11	115.8 (5)
C3—C2—H2A	121.0	C15—C10—C9	121.5 (5)
C4—C3—C2	122.1 (4)	C11—C10—C9	122.7 (5)
C4—C3—Cl1	118.3 (4)	C12—C11—C10	122.3 (5)
C2—C3—Cl1	119.6 (4)	C12—C11—H11A	118.8
C3—C4—C5	118.2 (4)	C10—C11—H11A	118.8
C3—C4—H4A	120.9	C11—C12—C13	119.7 (5)
C5—C4—H4A	120.9	C11—C12—H12A	120.2
C4—C5—C6	121.5 (4)	C13—C12—H12A	120.2
C4—C5—Cl2	116.7 (4)	C14—C13—C12	120.5 (5)
C6—C5—Cl2	121.8 (3)	C14—C13—H13A	119.8
C1—C6—C5	117.7 (4)	C12—C13—H13A	119.8
C1—C6—C7	118.2 (4)	C15—C14—C13	118.9 (5)
C5—C6—C7	124.1 (4)	C15—C14—H14A	120.5
O1—C7—C8	123.2 (4)	C13—C14—H14A	120.5
O1—C7—C6	118.4 (4)	C14—C15—C10	122.8 (5)
C8—C7—C6	118.4 (4)	C14—C15—Cl3	117.4 (4)
C9—C8—C7	120.9 (5)	C10—C15—Cl3	119.8 (4)
C9—C8—H8A	119.6		
			
C6—C1—C2—C3	−0.1 (7)	O1—C7—C8—C9	−11.5 (8)
C1—C2—C3—C4	−2.1 (7)	C6—C7—C8—C9	171.6 (5)
C1—C2—C3—Cl1	179.6 (4)	C7—C8—C9—C10	−179.7 (5)
C2—C3—C4—C5	2.9 (7)	C8—C9—C10—C15	−160.7 (5)
Cl1—C3—C4—C5	−178.8 (4)	C8—C9—C10—C11	19.5 (9)
C3—C4—C5—C6	−1.4 (7)	C15—C10—C11—C12	−1.5 (8)
C3—C4—C5—Cl2	−179.6 (4)	C9—C10—C11—C12	178.4 (5)
C2—C1—C6—C5	1.4 (7)	C10—C11—C12—C13	1.3 (9)
C2—C1—C6—C7	−179.1 (5)	C11—C12—C13—C14	−0.4 (9)
C4—C5—C6—C1	−0.6 (7)	C12—C13—C14—C15	−0.1 (8)
Cl2—C5—C6—C1	177.4 (4)	C13—C14—C15—C10	−0.1 (8)
C4—C5—C6—C7	180.0 (5)	C13—C14—C15—Cl3	179.6 (4)
Cl2—C5—C6—C7	−2.0 (7)	C11—C10—C15—C14	0.9 (8)
C1—C6—C7—O1	−44.2 (7)	C9—C10—C15—C14	−179.0 (5)
C5—C6—C7—O1	135.2 (5)	C11—C10—C15—Cl3	−178.8 (4)
C1—C6—C7—C8	132.8 (5)	C9—C10—C15—Cl3	1.3 (7)
C5—C6—C7—C8	−47.8 (7)		

### Hydrogen-bond geometry (Å, °)

**Table:**

*D*—H···*A*	*D*—H	H···*A*	*D*···*A*	*D*—H···*A*
C9—H9A···Cl3	0.93	2.66	3.042 (5)	106
C9—H9A···O1	0.93	2.53	2.841 (6)	100
